# Invariant natural killer T cells are functionally impaired in patients with systemic sclerosis

**DOI:** 10.1186/s13075-019-1991-y

**Published:** 2019-10-15

**Authors:** Ann-Christin Pecher, Felix Kettemann, Elisa Asteriti, Hannes Schmid, Silke Duerr-Stoerzer, Hildegard Keppeler, Joerg Christoph Henes, Reinhild Klein, Clemens Hinterleitner, Kathy-Ann Secker, Corina Schneidawind, Lothar Kanz, Dominik Schneidawind

**Affiliations:** 1 Centre for Interdisciplinary Clinical Immunology, Rheumatology and Autoinflammatory Diseases, University Hospital Tuebingen, Otfried-Mueller-Strasse 10, 72076 Tuebingen, Germany; 2 Department of Hematology, Oncology, Immunology, Rheumatology, Pulmonology, University Hospital Tuebingen, Otfried-Mueller-Strasse 10, 72076 Tuebingen, Germany

**Keywords:** Systemic sclerosis, Invariant natural killer T cells, Autoimmunity

## Abstract

**Background:**

Systemic sclerosis (SSc) is a potentially fatal autoimmune disease that leads to extensive fibrosis of the skin and internal organs. Invariant natural killer T (iNKT) cells are potent immunoregulatory T lymphocytes being able to orchestrate dysregulated immune responses. The purpose of this study was to evaluate numbers and function of iNKT cells in patients with SSc and to analyze their correlation with disease parameters.

**Methods:**

Human iNKT cells from 88 patients with SSc and 33 healthy controls were analyzed by flow cytometry. Their proliferative capacity and cytokine production were investigated following activation with CD1d ligand α-galactosylceramide (α-GalCer).

**Results:**

We observed an absolute and relative decrease of iNKT cells in patients with SSc compared with healthy controls. Interestingly, the subtype of SSc, disease severity, or treatment with immunosuppressive drugs did not affect iNKT cell numbers. However, T helper (Th) cell immune polarization was biased towards a Th17 immunophenotype in SSc patients. Moreover, iNKT cells from patients with SSc showed a significantly decreased expansion capacity upon stimulation with α-GalCer.

**Conclusion:**

iNKT cells are deficient and functionally impaired in patients with SSc. Therefore, adoptive transfer strategies using culture-expanded iNKT cells could be a novel approach to treat SSc patients.

## Background

Systemic sclerosis (SSc) is a rare connective tissue disease with an overall age- and sex-adjusted incidence rate of 5.6 cases per 100,000 person-years in the USA [1]. Its complex pathogenesis remains incompletely understood. It is acknowledged, though, that the pathogenesis of SSc is characterized by the following main features: first, vasculopathy; second, inflammation accompanied by the production of auto-antibodies; and third, tissue fibrosis through proliferation and differentiation of fibroblasts.

SSc can involve almost every organ, but affects especially the skin, lungs, heart, vessels, and gastrointestinal tract. Depending on the extent of skin involvement, patients are generally classified into two subtypes: limited cutaneous (lcSSc) and diffuse cutaneous SSc (dcSSc). These subtypes are prognostically relevant, as patients with dcSSc usually present with a more rapidly progressive fibrosis and early development of organ complications, resulting in a worse outcome than patients with lcSSc.

Unfortunately, treatment is usually organ based and non-curative [2]. Therefore, SSc has a high disease-related mortality, mostly attributed to pulmonary fibrosis [3], followed by pulmonary hypertension and cardiac involvement. To improve patients’ outcome, it is important to better understand the pathogenesis which might lead to targeted therapies.

This study focuses on a certain subset of T cells: invariant natural killer T (iNKT) cells, a subgroup of innate, non-MHC-restricted immune cells, which are CD1d restricted and express a semi-invariant T cell receptor (TCRα Vα14-Jα18 in mice and Vα24-Jα18 in humans) but also markers usually expressed by natural killer cells [4].

iNKT cells recognize selected endogenous as well as synthetic glycolipids like α-galactosylceramide (α-GalCer) and analogs by their TCR with high affinity and specificity [5, 6]. When activated, they typically release high amounts of pro- and/or anti-inflammatory cytokines [6]. The involvement of iNKT cells has been discussed in several autoimmune diseases [7–9]: in rheumatoid arthritis, diabetes, and systemic lupus erythematosus, a defect of iNKT cells has been observed.

In this study, we investigated iNKT cell numbers and function from patients with SSc. Due to their potent immunoregulatory properties, iNKT cells could be a promising target for novel cytotherapeutic approaches to treat autoimmune diseases such as SSc.

## Materials and methods

### Patients

This study was approved by the institutional review board of the Eberhard-Karls-University Tuebingen (IRB approval number 114/2016BO) to be in accordance with the ethical standards and with the Helsinki Declaration of 1975, as revised in 2013. From 2016 to 2017, peripheral blood samples of 88 patients with SSc were analyzed and compared to healthy controls (i.e., blood donors from the Center of Clinical Transfusion Medicine Tuebingen, *n* = 33). Patients were enrolled at the Centre for Interdisciplinary Clinical Immunology, Rheumatology and Autoinflammatory Diseases at the University Hospital Tuebingen, Germany.

### Isolation of PBMC

Peripheral blood samples were collected from SSc patients after written consent was obtained. First, peripheral blood mononuclear cells (PBMC) were isolated via Ficoll density gradient centrifugation (Biochrom) and cryopreserved in liquid nitrogen. Fresh PBMC were used for iNKT cell expansion experiments for both groups. For all other experiments, thawed PBMC were utilized.

### Multi-parametric flow cytometric analysis

Fresh or thawed PBMC were resuspended in staining buffer consisting of phosphate-buffered saline (PBS; Thermo Fisher Scientific) supplemented with 2% fetal bovine serum (FBS; Biochrom) followed by staining with fluorochrome-conjugated monoclonal antibodies (Ab). The following Ab were purchased from BD Biosciences or BioLegend: CD4 (RPA-T4), CD127 (A019D5), CD3 (OKT3), CD8 (HIT8a), CD19 (SJ25C1), and CD25 (BC96). To exclude dead cells, eBioscience™ Fixable Viability Dyes eFluor™ 506 and 780 (ThermoFisher Scientific) were used. Phycoerythrin (PE)-labeled PBS57-loaded CD1d tetramers were provided by the National Institutes of Health Tetramer Core Facility. Stained cells were measured using a LSR Fortessa flow cytometer (BD Biosciences), and analysis was performed with FlowJo 10.2 (Tree Star).

### Cytokine analysis

To analyze cytokine release through iNKT cell transactivation, 5 × 10^6^ PBMC from SSc patients or healthy donors were incubated in a 24-well plate together with 100 ng/ml α-GalCer (Sigma-Aldrich) in iNKT cell culture medium consisting of RPMI 1640 GlutaMAX™ Medium (ThermoFisher Scientific), 10% FBS (Biochrom), 100 U/ml PenStrep (Lonza), 5.5 μM 2-mercaptoethanol (Roth), 0.1 mM non-essential amino acids (NEAA; Gibco), 10 mM HEPES (Gibco), and 1 mM sodium pyruvate (Gibco).

To assess intracellular cytokines, cells were stimulated with 1x Cell Stimulation Cocktail (eBioscience) for 4 h at 37 °C and 5% CO_2_ in iNKT cell culture medium. After staining surface antigens, cells were fixed and permeabilized (eBioscience) prior to staining of intracellular interferon gamma (IFN-γ) (4S.B3, eBioscience), interleukin (IL)-4 (8D4-8, BioLegend), and IL-17 (A019D5, BioLegend). Stained cells were measured using a LSR Fortessa flow cytometer (BD Biosciences), and analysis was performed with FlowJo 10.2 (Tree Star).

### iNKT cell expansion assay

iNKT cells from SSc patients and healthy controls were expanded for 7 days from 2 × 10^6^ fresh PBMC per well in iNKT cell culture medium supplemented with 100 ng/ml α-GalCer (Sigma-Aldrich) and 100 IE/ml recombinant human interleukin 2 (rhIL-2; Novartis). IL-12 (R&D Systems) and CD19 magnetic-activated cell sorted B cells were added as indicated.

### Statistical analysis

Results are presented as mean ± standard deviation (SD). Data were compared using the Student’s *t* test. The Mann-Whitney *U* test was used for data that were not normally distributed. Correlations were investigated by calculating Pearson’s correlation coefficients. Differences with *p* values below 0.05 were considered statistically significant. Datasets were analyzed by SPSS Statistics version 24 (IBM) and Prism 7.03 (GraphPad Software).

## Results

### Patient characteristics

Eighty-eight patients with either lcSSc (65%) or dcSSc (35%) participated in our study. At enrolment, the median age was 53 years (range, 22–88) and median disease duration from the time of diagnosis was 8 years (range, 0–40). One third of the patients were pretreated with intensive immunosuppressive regimens such as cyclophosphamide (*n* = 23), rituximab (*n* = 2), or autologous stem cell transplantation (*n* = 6). Patients that underwent autologous hematopoietic cell transplantation within the last five years were excluded. Importantly, 42% of patients were off any immunosuppressive therapy at the time of blood draw for this study. The main clinical and laboratory characteristics are summarized in Table 1.

**Table 1:** Patients’ characteristics

	*n = 88*
Age—years	
Median	53
Range	22–88
Sex—no. (%)	
Female	66 (75)
Male	22 (25)
SSc subtype—no. (%)	
Limited cutaneous SSc	57 (65)
Diffuse cutaneous SSc	31 (35)
Disease duration—years	
Median	8
Range	0–40
Erythrocyte sedimentation rate—mm/h	
Mean	14
Range	0–94
C-reactive protein—mg/dl	
Mean	0.4
Range	0.01–2.52
Serum gamma globulins—%	
Mean	16
Range	9.4–38.3
Auto-antibodies—no. (%)	
Anti-nuclear Ab	82 (93)
Anti-Scl-70	39 (44)
Anti-centromere Ab	24 (27)
Modified Rodnan skin score (mRSS)	
Mean	8
Range	0–44
Pretreatment—no. (%)	
Cyclophosphamide	23 (26)
Rituximab	2 (2)
Autologous stem cell transplantation	6 (7)
Immunosuppressive therapy at blood draw—no. (%)	
None	37 (42)
Prednisolone	6 (7)
Cyclophosphamide	5 (6)
Mycophenolate	23 (26)
Azathioprine	5 (6)
Methotrexate	10 (11)
Others	3 (3)

### iNKT cell numbers are significantly reduced in patients with SSc

**Fig. 1:**
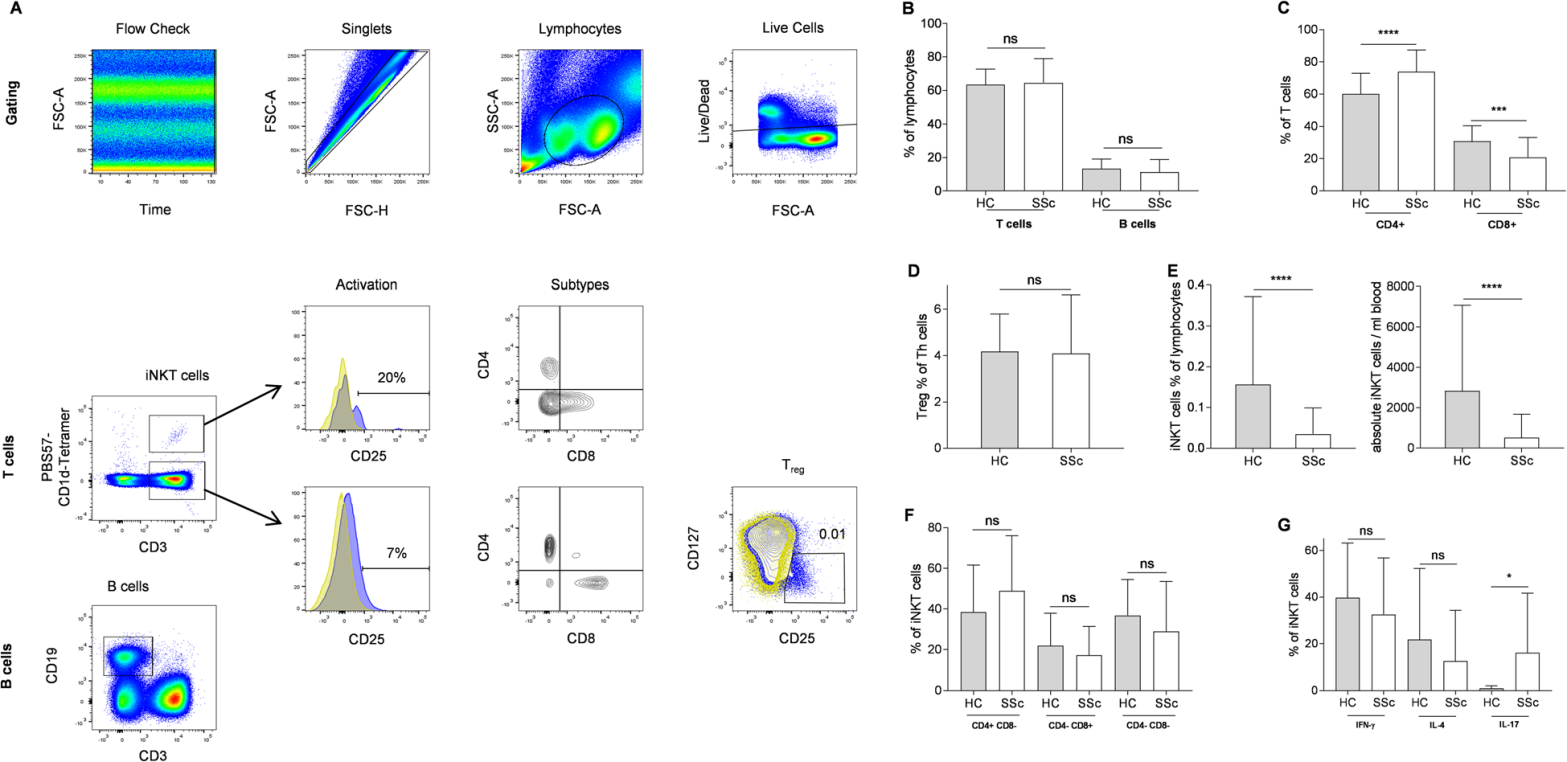
Gating strategy and different subsets of lymphocytes in healthy controls (HC) and patients with SSc. **a** Gating strategy that was applied in this study to quantify T cells, B cells, and Treg. iNKT cells were identified via CD3 and PBS57-CD1d-Tetramer staining. **b** T and B cells, **c** T cell subsets, **d** Treg (gated on CD4 Th cells), **e** iNKT cells, **f** iNKT cell subsets, and **g** intracellular cytokine staining for IFN-γ, IL-4, and IL-17 in iNKT cells from healthy controls and SSc patients. Absolute iNKT cell numbers per milliliter of blood were calculated as a percentage of total lymphocytes. Bars indicate SD. *p < 0.05, ***p < 0.001, ****p < 0.0001

Autoimmunity is driven by dysregulated lymphocytes against host antigens. We therefore determined conventional lymphocyte subsets and regulatory T cell subsets from SSc patients by flow cytometry. Our gating strategy is presented in Fig. 1a. We found comparable numbers of T cells and B cells in healthy controls and SSc patients (Fig. 1b). We did observe a predominance of CD4+ T helper (Th) cells with decreased CD8+ cytotoxic T cells in SSc patients (Fig. 1c). In contrast, numbers of CD4+CD25+CD127low regulatory T cells (Treg) were comparable with healthy controls (Fig. 1d). However, patients with SSc showed significantly lower relative and absolute numbers of iNKT cells in peripheral blood than healthy controls (Fig. 1e). Although CD4/CD8 iNKT cell subsets were equally distributed among both groups (Fig. 1f), intracellular cytokine staining revealed significantly increased numbers of IL-17-biased iNKT cells in SSc patients (Fig. 1g).

### iNKT cell numbers do not correlate with disease activity

**Fig. 2:**
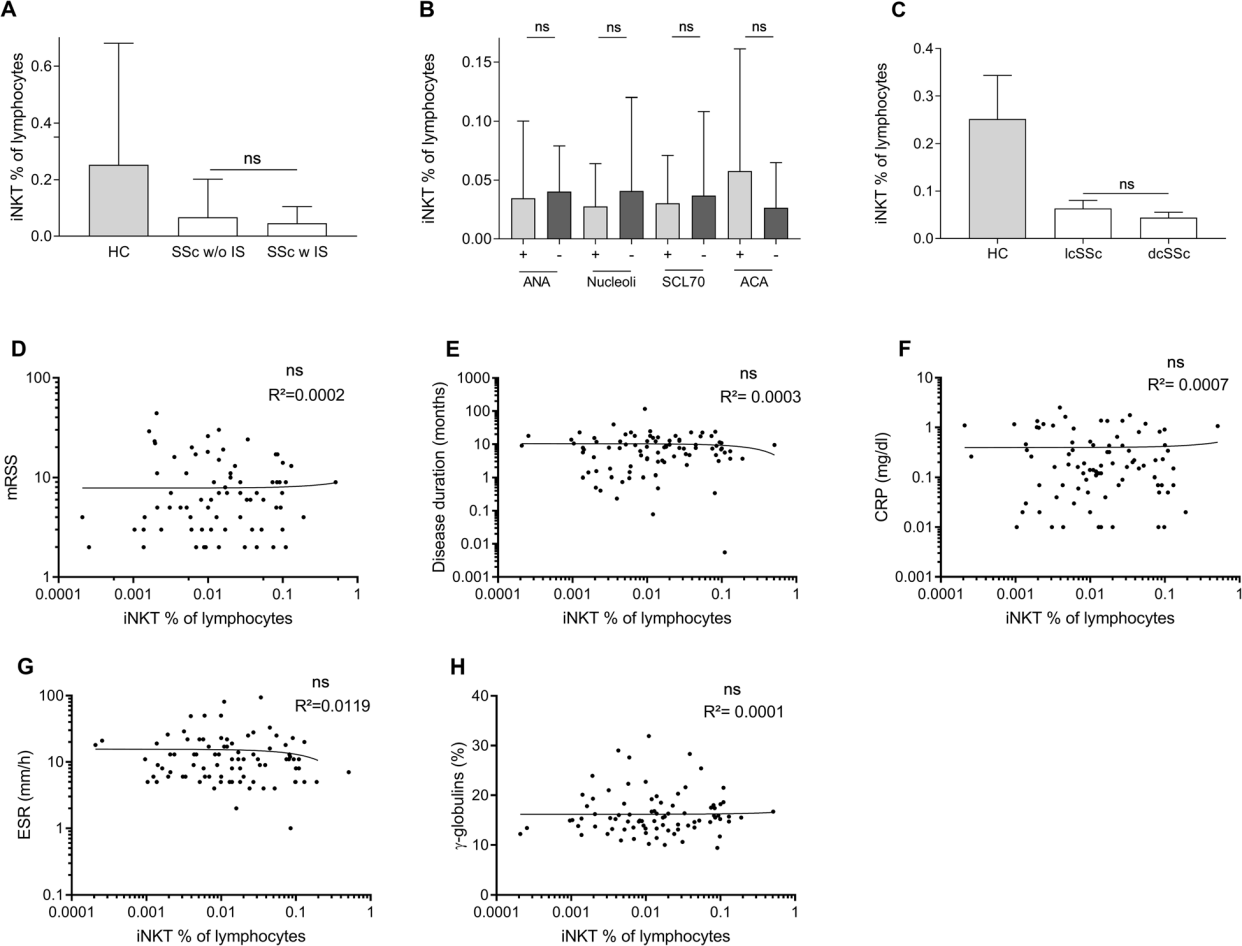
Comparison of different disease parameters and iNKT cells in patients with SSc. Impact of **a** immunosuppression and **b** SSc auto-antibodies at the time of diagnosis on iNKT cell numbers. Correlation of **c** the subtype of SSc, **d** the modified Rodnan skin score (mRSS), **e** disease duration, **f** C-reactive protein (CRP), **g** erythrocyte sedimentation rate (ESR), and **h** gamma globulins (γ-globulins) with iNKT cell numbers in SSc patients. Bars indicate SD

We also tested whether immunosuppressive therapy at the time of blood draw could have an impact on iNKT cell numbers in SSc patients. In fact, no significant difference in iNKT cell counts was found in SSc patients on immunosuppressive therapy compared with patients that did not take such drugs (Fig. 2a). This was also the case, when all patients after autologous stem cell transplantation were excluded.

In contrast to Treg, iNKT cells turned out to be significantly reduced in SSc patients. We therefore wondered whether well-established conventional disease activity parameters such as the modified Rodnan skin score (mRSS), the SSc subgroup (dcSSc/lcSSc), or inflammation markers correlate with iNKT cell numbers in the peripheral blood. The presence of auto-antibodies at the time of diagnosis did not significantly affect iNKT cell numbers (Fig. 2b). Moreover, we found no correlation between iNKT cells and mRSS, type of disease, disease duration, C-reactive protein (CRP), erythrocyte sedimentation rate (ESR), or gamma globulins (γ-globulins) (Figs. 2c–h).

### SSc Th cells are biased towards a Th17 phenotype

**Fig. 3:**
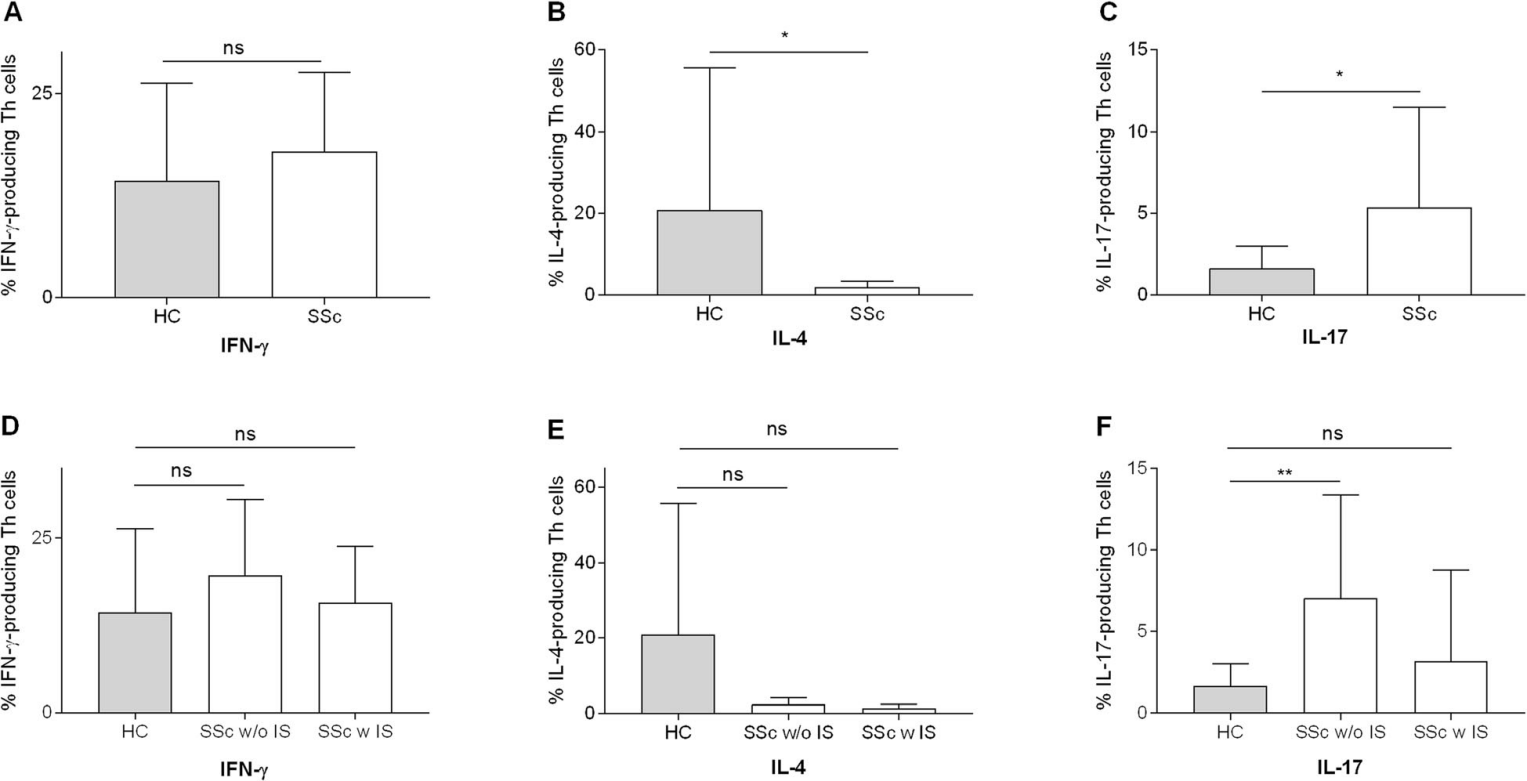
Intracellular cytokine staining for IFN-γ, IL-4, and IL-17 after 4 h of in vitro stimulation. **a** IFN-γ-producing, **b** IL-4-producing, and **c** IL-17-producing Th cells in healthy controls (*n* = 14) and SSc patients (*n* = 22). **d**–**f** SSc patients were stratified for immunosuppressive therapy at the time of blood draw. Bars indicate SD. *p < 0.05, **p < 0.01

iNKT cells are able to rapidly produce large amounts of immunoregulatory cytokines upon activation and thereby influence differentiation of Th cells into different phenotypes such as Th1, Th2, and Th17. We therefore performed an intracellular staining of the surrogate cytokines IFN-γ, IL-4, and IL-17, respectively. Our data demonstrate that IL-17-producing T cells are significantly increased in the peripheral blood of patients with SSc, whereas IL-4 production is significantly impaired (Fig. 3a–c). Interestingly, when treated with immunosuppressive medication, the increase of IL-17 production is partly reversed whereas no impact was noted on IL-4 and IFN-γ (Fig. 3d–f).

### Activation level of iNKT and Th cells in SSc

The IL-2 receptor α chain CD25 is one of the activation markers of conventional cytotoxic T cells, Th cells, and iNKT cells [10]. Our data illustrate that there was a comparable expression of CD25 on iNKT cells from SSc patients (*n* = 78) compared to healthy controls (*n* = 33): mean fluorescence intensity 323 (SD 319) for HC vs. 398 (SD 404) for SSc, *p* = 0.43; median fluorescence intensity 170.5 (range 1112) for HC vs. 285.5 (range 2531) for SSc, *p* = 0.35.

### iNKT cells from SSc patients fail to expand upon stimulation

**Fig. 4:**
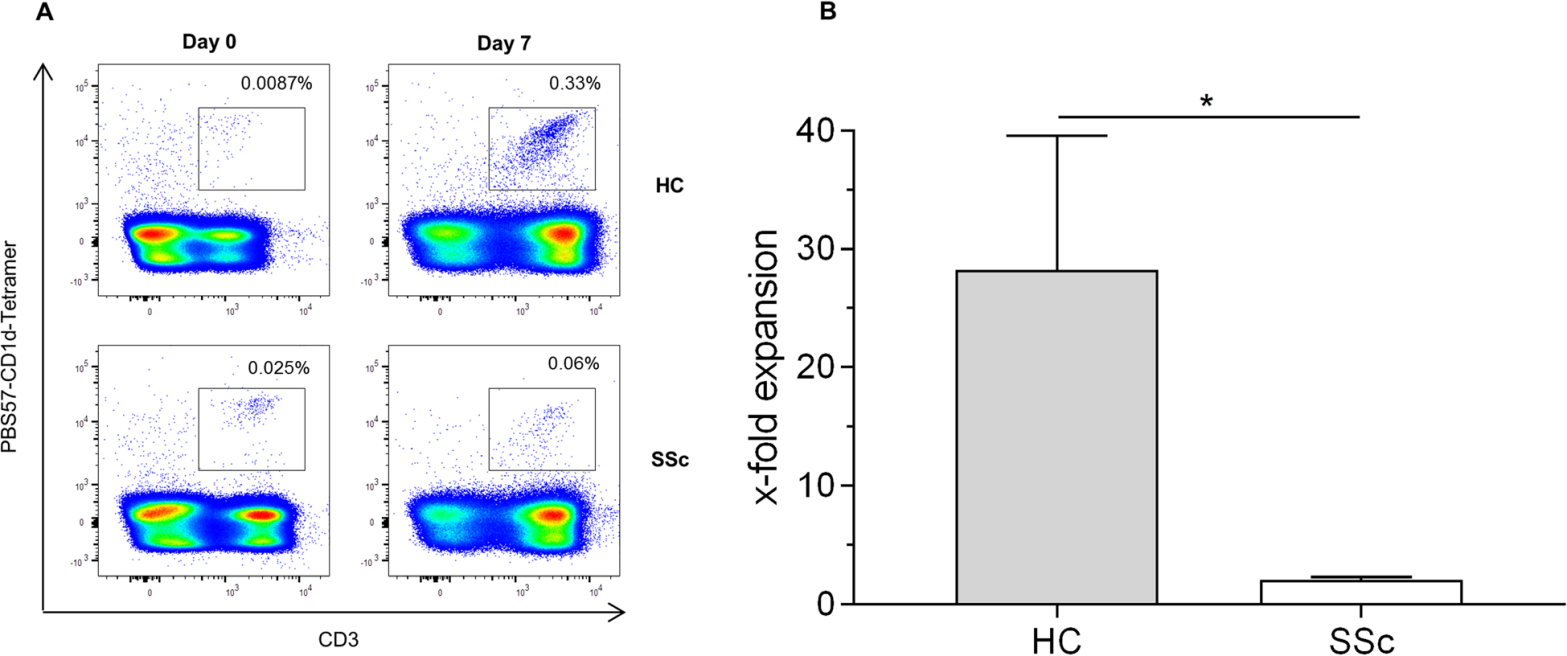
Expansion capacity of iNKT cells. **a** Representative dot plots and **b** fold expansion of fresh iNKT cells 7 days after ex vivo culture of PBMC from healthy controls (*n* = 4) and SSc patients (*n* = 4) with α-GalCer and rhIL-2. Bars indicate SD. *p < 0.05

To further evaluate the functionality of iNKT cells, we tested their ability to expand upon stimulation with the glycolipid ligand α-GalCer and IL-2. We used fresh PBMC for this experiment in both groups to exclude cellular alterations and impacts on cell proliferation by cryopreservation. After 7 days of cell culture, iNKT cells from healthy controls could be expanded 28-fold (Fig. 4). In contrast, iNKT cells from SSc patients did scarcely proliferate.

**Fig. 5:**
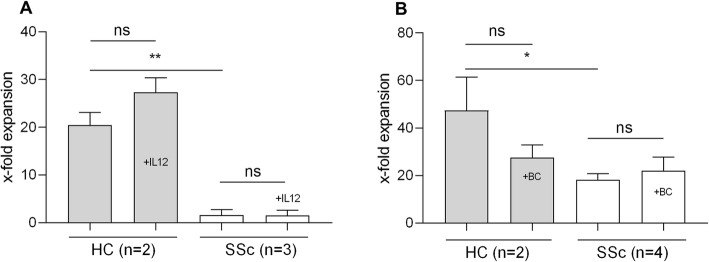
Expansion capacity of iNKT cells with IL-12 and healthy antigen-presenting cells. Fold expansion of iNKT cells after 7 days of ex vivo culture of PBMC from healthy controls and SSc patients with α-GalCer, rhIL-2, and **a** ± 50 ng/ml IL-12 (HC 20.5 ± 2.7 versus 27.4 ± 3.0, SSc 1.6 ± 1.2 versus 1.5 ± 1.1) or **b** ± 200,000 healthy B cells (BC) (HC 47.4 ± 14.0 versus 27.5 ± 5.3, SSc 18.2 ± 2.5 versus 22.1 ± 5.6) per well. Bars indicate SD. *p < 0.05, **p < 0.01

Neither addition of IL-12 (Fig. 5a) nor coculture with healthy antigen-presenting B cells (BC; Fig. 5b) could ameliorate the proliferation defect of SSc patient iNKT cells. This observation supports our hypothesis of an intrinsic iNKT cell dysfunction in SSc patients.

An impaired proliferative capacity of iNKT cells could explain our previous finding of significantly reduced iNKT cell numbers and why these cells fail to induce immune tolerance in SSc patients.

## Discussion

Autoimmune diseases such as SSc are characterized by a breach of immune tolerance against auto-antigens resulting in inflammation and fibrosis. iNKT cells are a subset of T lymphocytes that harbor incredible immunoregulatory properties through the instant release of cytokines upon stimulation with glycolipids. We could show previously that adoptive transfer strategies using iNKT cells protect from graft-versus-host disease in murine models of allogeneic bone marrow transplantation [11]. This clearly indicates that iNKT cells are able to maintain and restore immune tolerance.

Therefore, we investigated the role of iNKT cells in SSc patients. In this study, we demonstrate that iNKT cells are significantly reduced in the peripheral blood of patients with SSc compared to healthy controls. This finding correlates with data from other autoimmune diseases where iNKT cell numbers were diminished [7, 12]. However, we did not observe a significant correlation between disease or activity parameters as previously described by Riccieri et al. on 50 patients with SSc [13]. As the number of iNKT cells does not correlate with disease duration or severity, we assume that iNKT cell deficiency might be critical in the early phases of SSc pathogenesis, contributing to or being a result of an immune tolerance breach. The importance of iNKT cells in the early stage has been shown for other autoimmune diseases, where the early activation of iNKT cells already resulted in an amelioration of autoimmunity [14]. Importantly, immunosuppressive therapy at the time of blood draw did not influence iNKT cell numbers excluding a potential bias through lymphocytotoxic effects.

It is not fully elucidated yet, how iNKT cells interact with other parts of the immune system in SSc. However, they produce immunoregulatory cytokines influencing immune cell differentiation. The IL-23-Th17 axis seems to present an important pathway in the development of autoimmune diseases [15]. Like other authors [16], we could demonstrate a Th17 immune bias in the peripheral blood of patients with SSc. IL-17 has been suggested to play an important role in the induction and maintenance of SSc through its potential pro-fibrotic and inflammatory signaling [17], but its pathophysiological role is still a matter of debate. Interestingly, this effect can be reversed when patients were treated with immunosuppressive drugs.

Furthermore, the ability of iNKT cells to respond and proliferate upon stimulation with α-GalCer is significantly lower in patients with SSc than in healthy controls which suggests a critical functional deficit that could explain low iNKT cell numbers in SSc patients. Interestingly, this defect was not improved by costimulation with healthy B cells or addition of IL-12. Hyporeactive iNKT cells may lack the ability to induce a tolerogenic Th cell polarization and, therefore, fail to inhibit proinflammatory activities of the immune system.

Low numbers and functional defects of iNKT cells in SSc patients might constitute a therapeutic target: adoptive transfer strategies could contain dysregulated immune responses and restore immune tolerance in patients with autoimmune diseases. To our knowledge, this is the largest study done on iNKT cells in patients with SSc. Our data strongly suggest that iNKT cells and their role in SSc pathogenesis warrant further investigation to develop a novel therapeutic approach to cure autoimmune diseases such as SSc.

## Conclusions

iNKT cells are deficient and functionally impaired in patients with SSc. Therefore, adoptive transfer strategies using culture-expanded iNKT cells could be a novel approach to treat SSc patients.

## Data Availability

The datasets used and/or analyzed during the current study are available from the corresponding author on reasonable request.

## Abbreviations

α-GalCer: α-Galactosylceramide
Ab: Antibodies
ACA: Anti-centromere antibodies
ANA: Anti-nuclear antibodies
Nucleoli: Anti-nucleoli antibodies
SCL70: Anti-topoisomerase I antibodies
BC: B cells
Treg: CD4+CD25+CD127low regulatory T cells
CD: Cluster of differentiation
CI: Confidence interval
CRP: C-reactive protein
dcSSc: Diffuse cutaneous SSc
DN: Double negative
ELISA: Enzyme-linked immunosorbent assay
ESR: Erythrocyte sedimentation rate
FSC: Forward scatter
γ-globulins: Gamma globulins
DGRh: German Society for Rheumatology
HC: Healthy controls
IFN-γ: Interferon gamma
IL: Interleukin
iNKT cells: Invariant natural killer T cells
IS: Immunosuppressive medication
lcSSc: Limited cutaneous SSc
mRSS: Modified Rodnan skin score
ns: Not significant
PBMC: Peripheral blood mononuclear cells
rhIL-2: Recombinant human IL-2
SSC: Side scatter
SEM: Standard error of the mean
SSc: Systemic sclerosis
TCR: T cell receptor
Th cells: T helper cells
w: With
w/o: Without
